# Inactivation of DAP12 in PMN Inhibits TREM1-Mediated Activation in Rheumatoid Arthritis

**DOI:** 10.1371/journal.pone.0115116

**Published:** 2015-02-02

**Authors:** Xianghong Chen, Erika A. Eksioglu, John D. Carter, Nicole Fortenbery, Sarah S. Donatelli, Junmin Zhou, Jinhong Liu, Lili Yang, Danielle Gilvary, Julie Djeu, Sheng Wei

**Affiliations:** 1 Department of Immunology, H. Lee Moffitt Cancer Center, Tampa, Florida, United States of America; 2 Department of Internal Medicine, Division of Rheumatology, University of South Florida College of Medicine, Tampa, Florida, United States of America; 3 Department of Immunology, Tianjin Medical University Cancer Hospital and Research Institute, Tianjin, China; Institute of Hepatology - Birkbeck, University of London, UNITED KINGDOM

## Abstract

Rheumatoid arthritis (RA) is an autoimmune disease characterized by dysregulated and chronic systemic inflammatory responses that affect the synovium, bone, and cartilage causing damage to extra-articular tissue. Innate immunity is the first line of defense against invading pathogens and assists in the initiation of adaptive immune responses. Polymorphonuclear cells (PMNs), which include neutrophils, are the largest population of white blood cells in peripheral blood and functionally produce their inflammatory effect through phagocytosis, cytokine production and natural killer-like cytotoxic activity. TREM1 (triggering receptor expressed by myeloid cells) is an inflammatory receptor in PMNs that signals through the use of the intracellular activating adaptor DAP12 to induce downstream signaling. After TREM crosslinking, DAP12’s tyrosines in its ITAM motif get phosphorylated inducing the recruitment of Syk tyrosine kinases and eventual activation of PI3 kinases and ERK signaling pathways. While both TREM1 and DAP12 have been shown to be important activators of RA pathogenesis, their activity in PMNs or the importance of DAP12 as a possible therapeutic target have not been shown. Here we corroborate, using primary RA specimens, that isolated PMNs have an increased proportion of both TREM1 and DAP12 compared to normal healthy control isolated PMNs both at the protein and gene expression levels. This increased expression is highly functional with increased activation of ERK and MAPKs, secretion of IL-8 and RANTES and cytotoxicity of target cells. Importantly, based on our hypothesis of an imbalance of activating and inhibitory signaling in the pathogenesis of RA we demonstrate that inhibition of the DAP12 signaling pathway inactivates these important inflammatory cells.

## Introduction

Rheumatoid arthritis (RA) is a relatively common autoimmune disease affecting over two million people in US and characterized by dysregulalted and chronic systemic inflammatory responses that affect the synovium, bone, and cartilage causing damage to extra-articular tissue [1, 2]. Its progression is also chronic and debilitating due to which patients experience perpetual pain and, as the disease progresses, major deformation. Among the many inflammatory cells that play a role in the development of the pathogenesis of this disease are innate immune cells, such as macrophages, monocytes and neutrophils [3]. The last group, neutrophils, are major players in innate immunity and the first line of defense against invading pathogens by assisting in the initiation of adaptive immune responses (Reviewed in [4]). Also called, polymorphonuclear leukocytes (PMNs) due to the appearance of multiple nuclei, they comprise the largest population of white blood cells in the peripheral blood as it accounts for about 40–70% of the total white blood cell population during inflammatory stimulation [4, 5]. They perform their main innate immune function through phagocytosis, cytokine production and their NK-like activity.

Activated PMNs initiate their function regulated by a balance of inhibitory and activating signals emanating from receptors, containing the functional motifs immune-receptor tyrosine-based inhibitory motif (ITIM) and immunoreceptor tyrosine-based activating motif (ITAM) respectively, that aid in the recruitment of molecules that guide their signaling cascade [6, 7]. Activating receptors are involved in the activation of direct lysis and/or cytokine production in immune effector cells through the recruitment of adaptor proteins, such as DNAX activation protein of 12 kDa (DAP12), which then in turn recruits the kinase Syk70 leading to the activation of the downstream molecules PI3K and ERK. Importantly, RA patients are known to have an increased expression of DAP12 which appeared predominantly expressed in cells of myeloid origin including mononuclear cells in lymphocyte aggregates, including myeloid cells like macrophages [8, 9]. Under normal circumstances, inhibitory receptors phosphorylate their ITIM domains, after engagement with their ligand, which provides a binding site for phosphatases that inhibit downstream signaling. However, in abnormal inflammatory conditions, such as transformation or stress, activating receptors bind to their ligand recruiting ITAM containing adaptors, such as DAP12, which become phosphorylated and in turn recruit kinases like Syk and PI3K, leading to the activation of ERK signaling [8, 9].

In recent years it has been identified that PMNs contain a family of activating receptor called triggering receptor expressed by myeloid cells (TREM) [10]. Currently, at least five members of the TREM family have been identified (TREM1, TREM2, TREM3, TREM4 and TREM5) with TREM1, mainly present in monocytes and neutrophils, categorized as playing a very important role in acute and chronic inflammatory responses [11–13]. Importantly, TREMs use exclusively DAP12 as their ITAM adaptor through a positively charged lysine in their trans-membrane domain that complements DAP12’s negative charge at its trans-membrane domain [13]. After TREM crosslinking, the DAP12 ITAM tyrosines are phosphorylated providing an open binding site for Syk tyrosine kinases with which to lead the activation of PI3 kinase, phospholipase Cc (PLCc) and extracellular signal-related kinase (ERK) 1/2 with a concomitant increase in intracellular calcium and activation of NF-κB signal pathways critical for maintenance of the inflammatory response [14].

In these studies we tested the hypothesis that disruption of the balance between inhibition and activation contributes to the pathogeneses of RA and causes tissue damage. Our results confirm the important role of both TREM1 and DAP12 expression, specifically in neutrophils, in the pathogenesis of RA and demonstrate that blocking DAP12 reduces the activation of these cells which could serve as a tool to study pathogenic tissue damage.

## Methods

### Isolation of neutrophils from blood and synovial fluid

Healthy donor blood was obtained from Florida Blood Services and RA synovial fluid samples came from the University of South Florida IRB #: 6532. Written consent was received from all patients for whom samples were collected. Peripheral blood mononuclear cells (PBMC) were isolated by Ficoll-Paque (Amersham Biosciences, Uppsala, Sweden) gradient by diluting 1:2 the specimens in 1X PBS and centrifuging at 1600 rpm for 30 minutes. Neutrophils, the layer of white blood cells immediately above the red blood cells, were isolated and washed in PBS (Mediatech, Inc. Herndon, VA) three times, then resuspended in RPMI 1640 medium (Mediatech, Inc.) supplemented with 10% FBS (Atlanta Biologicals, Lawrenceville, GA) and 2% PSG (Mediatech, Inc., hereon referred to as complete RPMI). After isolation cells were washed twice with PBS and cultured for 1.5 hours in complete RPMI at 37°C, 5% CO_2_ followed by passing non-adherent cells through a ninole filter to separate NK cells from T-Cells. Similarly, RA synovial fluid was diluted in 1X PBS and spun down at 400g for 20 minutes prior to Ficoll isolation.

### Transfection of TREM1 and DAP12

Plasmid vectors for TREM-1, wild type DAP12 or double negative (dn) DAP12 were transfected into the human embryonic kidney cell HEK293 (ATCC CRL-1573) with lipofectamine transfection reagent following the manufacturer’s instructions (Invitrogen). After 36 hours, cells were cultured with an anti-TREM-1 monoclonal antibody (R & D systems) at room temperature for 20 minutes, followed by three 1X PBS washes and cross-linked with rabbit anti-mouse IgG (R & D systems, Minneapolis, MN) at 37°C for 10 minutes before harvesting the cells for analysis. In crosslinking experiments, HEK293 cells were washed with PBS and re-suspended in 2.0 µg/ml of anti-TREM-1 antibody on ice for 30 min.

### Western blot analyses

Freshly purified PMN cells from RA patients or healthy blood donors were used for Western blot analysis. Briefly, after washing with PBS, cells were incubated with 1.0 µg /ml of Affinipure rabbit anti-mouse IgG (H+L) Ab (Jackson Immunoresearch) at 37°C for 10 min. Lysates were prepared by resuspending the cell pellet in 1% NP-40, 10 mM Tris, 140 mM NaCl, 0.1 mM PMSF, 10 mM iodoacetamide, 50 mM NaF, 1 mM EDTA, 0.4 mM sodium orthovanadate, 10μg/ml leupeptin, 10 μg/ml pepsatin, and 10μg/ml aprotinin and lysing on ice for 30 min. Cell lysates were centrifuged at 12,000g for 15 min to remove nuclei and cell debris. The protein concentration of the soluble extracts was determined by using the Bio-Rad (Bradford) protein assay and 50 μg of protein was detected by Western blotting with anti- anti–phosphorylated-ERK1/2 (New England Biolabs, Beverly, MA), or anti–phosphorylated-AKT (Cell Signaling Technology). Equal loading was assessed by reblotting Anti–total ERK, and anti–total AKT antibodies (Cell Signaling Technology, Danvers, MA). Proteins were detected with the enhanced chemiluminescence detection system (Amersham ECL, GE Healthcare Bio-Sciences).

### Quantitative Real-time-RT-PCR (qPCR) for neutrophils

Total RNA from the neutrophils was isolated using Trizol-Reagent (life technologies, Grand Island, NY) according to the manufacturer’s protocol. Human TREM-1 and DAP12 mRNA expression was assessed by first-strand cDNA synthesis from 1ug of total RNA. A reverse transcription reaction was performed using iScript cDNA Synthesis Kit (Bio-Rad, Hercules, CA) with a final volume of 20ul. Incubation periods were as follows: 5 minutes at 25°C, 30 minutes at 42°C, 5 minutes at 85°C and hold at 4°C. Afterwards, quantitative RT–polymerase chain reaction (qRT-PCR) reactions were performed by iQ SYBR Green Supermix of Bio-Rad according to the manufacturer’s recommendation with 1.5ul of cDNA. A negative control without cDNA template was run with every assay. Oligonucleotide primers were obtained through IDT, Inc.: TREM-1 forward 5’- GTG GTG ACC AAG GGT TTT T -3’ and reverse 5’- ACA AGG CCT TAG TGG TGG T -3’; DAP12 forward 5’-GAG ACC GAG TCG CCT TAT C-3’ and reverse 5’-ATA CGG CCT CTG TGT GTT G-3’; GAPDH forward 5’-GAA GGT GAA GGT CGG AGT -3’ and reverse 5’-GAA GAT GGT GAT GGG ATT TC-3’. Conditions were 50°C for two minutes, 95°C for 15 min, then 40 cycles of 95°C for 15 seconds and 60°C for one minute. The fluorescence was measured with a Q-PCR machine (Bio-Rad) at every cycle. Transcript copy number per subject was calculated by normalization to GAPDH expression.

### Surface phenotyping by flow cytometry

Determination of TREM-1 expression on PMN, NK cells, T-cells and monocytes was carried out using both healthy and RA primary cells by flow cytometry with anti–CD66, CD3, CD56 and CD14 respectively, as well as anti–TREM-1 antibody (BD Biosciences, San Jose, California). Data acquisition and analysis was carried out on a FACScalibur flow cytometer (BD Biosciences) using the Cell Quest software program (BD Biosciences). Neutralization experiments were carried out using an anti- tumor necrosis factor-α (TNFα) blocking antibody (Biolegend) following the manufacturer’s recommendation prior to assessment of TREM1 and DAP12 (SantaCruz) by flow cytometry.

### Cytotoxicity assays

Cytotoxicity assays were performed through the release of chromium from target labeled cells. Briefly, a ^51^Cr-release assay was performed using HTB-93, Human synovial cell line as targets for PMN effector cells. Briefly, 1×10^6^ target tumor cells were labeled with 100 µCi of Na [^51^Cr] chromate (Amersham Corp.) in 0.5 ml of medium at 37°C in a 5% CO_2_ atmosphere for 1 hour. The cells were then washed three times and added to effector cells at 5 × 10^3^ cells/well in 96-well round-bottomed microplates, resulting in E/T ratios ranging from 50:1 to 6.25:1 in a final volume of 0.2 ml per well. After 5 h incubation at 37°C, 100 µl of culture supernatants was harvested and counted in a gamma counter. The percent specific ^51^Cr release was determined according to the equation [(experimental cpm-spontaneous cpm)/total cpm incorporated] × 100. All determinations were done in triplicate.

### Vaccinia viral delivery of dominant negative DAP12

Expression cassettes encoding FLAG-tagged human DAP12 and MEK were constructed in the pLF plasmid (a derivative of pcDNA3; Invitrogen) and mutagenized with the QuickChange Site Directed Mutagenesis Kit (Stratagene, La Jolla, CA) to create dominant negative DAP12 and MEK. These constructs were later transferred to a pSP11 vector from which the recombinant viruses were constructed of the WR strain of vaccinia. Cells were then further incubated in serum-containing medium for 4 h at 37°C. CD56 expressing vaccinia was used as a control for nonspecific effects of viral infection. For infection, neutrophils were incubated with the vaccinia constructs for 2 hours at 37°C in serum-free medium at a multiplicity of infection (MOI) of 5. Cells were then further incubated in serum-containing medium for 4 h at 37°C.

### ELISA

Cell-free primary RA synovial fluid and plasma from healthy blood donors were used for detecting IL6 (BD OptEIA), IL8 (BD OptEIA), IL-17 (peptrotech), TNFα (Pierce-endogen) and RANTES (Ray Biotech) by the BD OptEIA enzyme-linked immunosorbent assay (ELISA) Kit following the manufacturers’ protocol. Briefly, non-tissue culture treated 96-well plates (Becton Dickson) were coated with capture antibody diluted in coating buffer (eBioscience) and incubated overnight at 4°C. PBS (Mideatech, Inc.) 10% FBS (Atlanta Biologicals) was used for blocking and as assay diluent as specified in the protocol. All washes during the protocol were performed with a Bio-Rad wash machine (Model 1575 Immunowash) using PBS with 0.5% Tween-20 (Sigma). After adding substrate, a color reagent TMB substrate also from BD biosciences, samples were stopped with the TMB stop solution (Kirkegaard and Perry Labs). Plates were read in a Spectramax PLUS 384 spectrophotometer at 450 nm with a background subtraction wavelength of 570 nm.

### Immunostaining

PMN cells from healthy donors and from the synovial fluid of RA patients were pretreated with dnDAP12 for 1 h at 37°C and incubated with HTB-93 cells at a 1:1 ratio in a total volume of 100 µl. The cells were spun at 1000 rpm for 1 min at 4°C in a microcentrifuge and incubated for 0–10 min at 37°C. Untreated or CD56-treated PMN cells were included as controls. Cell-Tracker Orange (Molecular Probes, Eugene, OR) was used to pre-stain HTB-93 cells followed by immunostaining. Cells were centrifuged onto a microscope slide, fixed at-20°C with methanol/acetone (3/1) for 20 min, air-dried and rehydrated for 2 h in changes of triton 100 buffer for 5 minutes and 10 minutes at room temperature. A monoclonal anti-human granzyme B Antibody (Research Diagnostics, North Las Vegas, NV) diluted 1/200 in 1% BSA in PBS, was applied to the slides for 1 h followed by 1X PBS washes before incubation with secondary goat anti-mouse Ig FITC-labeled Ab (1/100 dilution Sigma-Aldrich, St Louis, MO) for 30 min. Samples were viewed with a Leica DMLB upright fluorescence microscope with a 100*/1.3 NA oil-immersion objective (Leica Microsystems, Germany), and DAPI, FITC, and Texas Red filter cubes. Images were produced using the Retiga 1300 CCD camera (QIamging, Surrey, BC) and IPlab version 3.1 software suite (BD Bioscience Imaging, Rockville, MD). On each slide 100 PMN/HTB-93 conjugates were evaluated for granzyme B mobilization.

### Statistical Analysis

Statistical analysis was performed using Student’s t test, and values of p < 0.05 were considered significant. All experiments were performed more than three independent for each primary specimen. The data represent the mean value of triplicates ± SEM.

## Results

**Figure 1:**
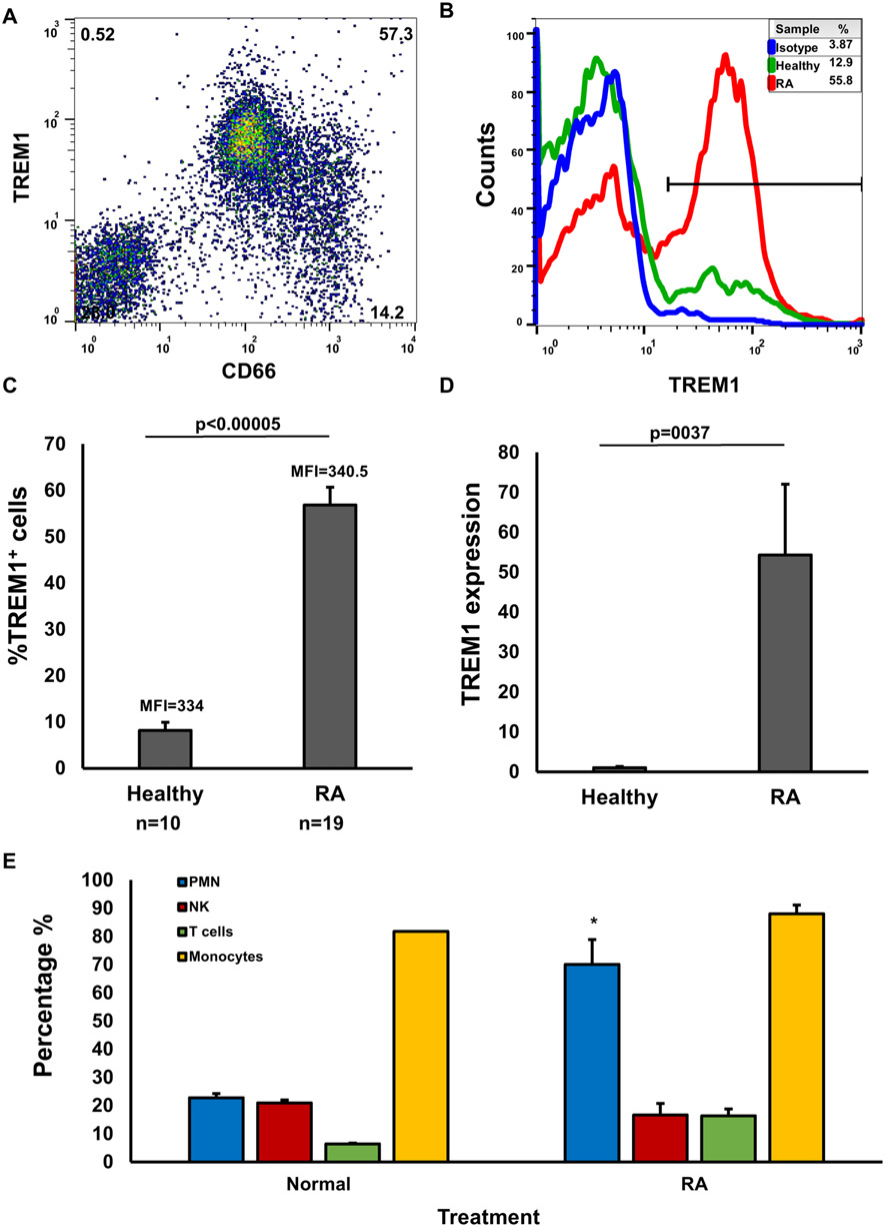
Increased expression of TREM-1 in PMN from RA patients. (A) PMN cells isolated from the synovial fluid of RA patients and healthy donors were analyzed by flow cytometry for the cell surface expression of TREM-1. A representative dot plot of CD66/TREM-1 expression is shown. (B) Representative figure of the expression of TREM-1 on the isotype stained cells, healthy PMN and the PMN from a primary RA specimen analyzed by flow cytometry. Legend shows the percent of total TREM 1 cells from the gate shown. (C) The same analysis shown in B was used to determine the relative levels of TREM-1 in isolated PMN from RA primary specimens compared with healthy controls (n = 19 RA and n = 6 controls). The MFI of all the samples was also obtained and averaged. However there were not significant changes (numbers shown). (D) Quantitative RT-PCR was used to determine the relative levels of TREM-1 in isolated PMN from RA primary specimens compared with healthy controls (n = 19 RA and n = 6 controls). (E) Surface expression of TREM-1 was compared between PMNs (blue), NK cells (red), T cells (green) and monocytes through flow cytometric analysis of isolated (n = 6). Error bars denote the standard error of duplicate determinations of 5 samples per group and p values are shown between figures except in E where it is shown as an asterisk to denote p = 0.0013 of normal versus RA while the other ones were not significant.

While the expression of TREM1 in non-infectious inflammatory conditions like RA is known to be up-regulated [15–19], the status of this molecule in PMNs has not been previously explored. We started by examining the expression of TREM-1 in the PMNs of RA primary specimens, after isolating granulocytes by Ficoll gradient centrifugation and using CD66 as a specific flow cytometric marker for these cells (Fig. 1A). Based on a flow cytometric analysis as shown in Fig. 1B, over 50% of these cells expressed TREM1 with a significantly higher expression in the PMNs of RA patients compared to much lower levels in normal donor controls (Fig. 1C, RA n = 19 and control n = 6). While this increase was also matched with an increase in TREM1 gene expression from the sorted PMNs of RA patients by real-time RT-PCR (Fig. 1D), it did not correlate with an increase in surface receptor expression per cell as the MFI levels remained similar and were not statistically different (average values shown in Fig. 1C). Importantly, TREM1 expression was not increased in other of the cells we tested including NK cells, T cells and monocytes compared to controls (Fig. 1E, *p<**0.05**) and the levels of their MFI also had no significant differences (not shown) just as we saw with PMNs (Fig. 1C).

**Figure 2:**
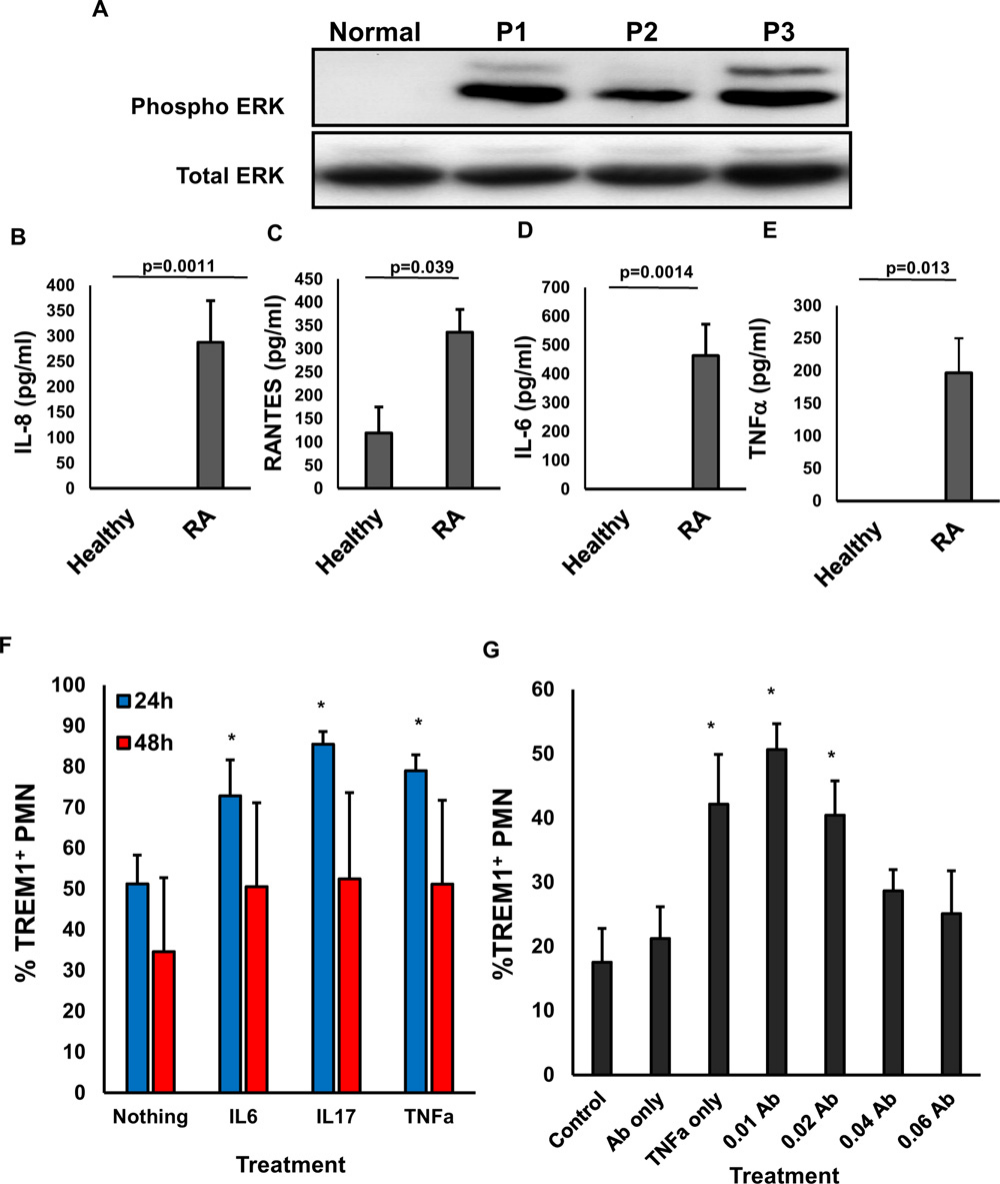
PMN cells from RA patients maintain constitutively activated ERK1/2. (A) PMN cell lysates were prepared from RA patients and healthy donors and used to measure the activity of ERK by Western blot using antibodies specific for: phosphorylated or total ERK1/2. Concentrations of IL-8 (B), RANTES (C), IL-6 (D) and TNFα (E) were determined by ELISA from either the synovial fluid of RA patients or the serum of healthy donors. Error bars denote the SEM triplicate determinations of n = 19 RA samples and n = 6 controls, p values as shown. (F) Healthy PMN (n = 4) were isolated and cultured in the presence of either TNFα (1ng/ml), IL-6 (1ng/ml), or IL-17 (20ng/ml) for 24 and 48 hours before TREM1 analysis by flow cytometry (G) Healthy PMN (n = 4) were isolated and cultured in the presence of TNFα (0.01ug/ml to 0.1ug/ml) after the addition of TNFα blocking antibody for 24 and 48 hours before TREM1 analysis by flow cytometry. For both F and G error bars denote the SEM of duplicate readings of 4 treated primary healthy specimens. Asteriks denote p<0.05 against control untreated cells.

To assess the functional activation status of TREM-1 on these cells, we tested the activation of ERK in PMNs isolated from RA patients as well as the secretion of the RA-associated inflammatory cytokines involved in PMN activation [20, 21]. We found a very high ERK activation in RA patients, as detected by western blot of phosphorylated ERK, without changes in the levels of total ERK (Fig. 2A). Similarly, the level of the cytokines: IL-8 (Fig. 2B), RANTES (Fig. 2C), IL-6 (Fig. 2D) and TNFα (Fig. 2E) were significantly elevated, suggesting that the up-regulation of TREM1 correlates with activation of PMNs in RA patient specimens and demonstrating that TREM1 increased expression is also linked to an increase in its activity. It is well known that pro-inflammatory cytokines, such as TNFα, IL-6, and IL-17 are crucial mediators in rheumatoid synovitis and subsequent bone destruction in RA [21] and our data shows a correlation with excess cytokine secretion and TREM1 expression. For this reason we tested the role of these cytokines in modulating the expression of this receptor in healthy PMNs. After 24 hours of culture, PMNs treated with human recombinant versions of TNFα (1ng/ml), IL-6 (1ng/ml), and IL-17 (20ng/ml) significantly increased the expression of TREM1, although this increase was not sustained as it was not significant by 48 hours of culture (Fig. 2F). Interestingly, TNFα induced an increase in TREM1 expression in healthy PMNs which made us wonder if blocking it, a commonly used strategy for RA, could prevent its up-regulation in these cells. Healthy PMNs were treated with recombinant human TNFα (1ng/ml) and increasing doses of a blocking TNFα antibody (neutralization ND50 was reported to be between 0.01–0.06 ug/ml according to the manufacturer’s recommendation, Biolegend). As shown in Fig. 2G, blocking TNFα induced a dose-dependent reduction in the expression of TREM1 stimulated by the cytokine.

**Figure 3:**
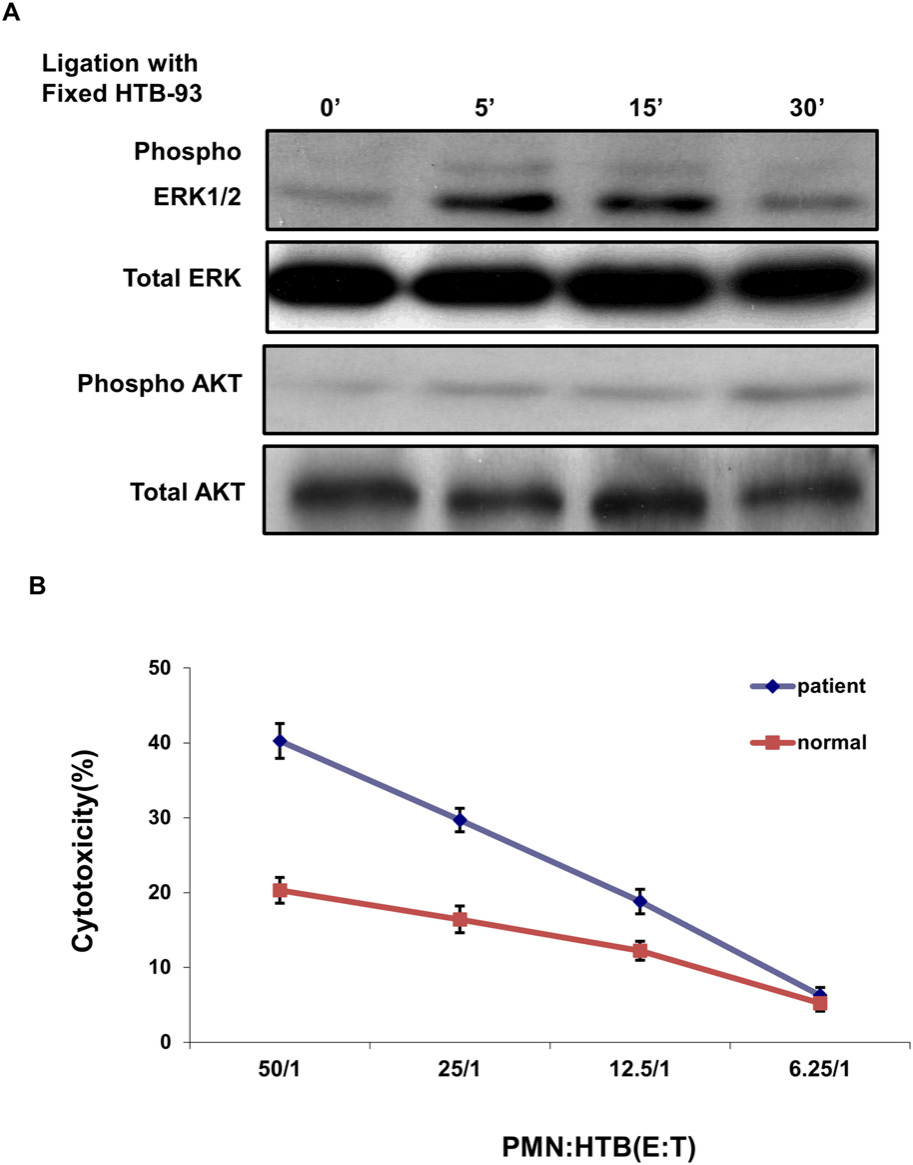
Increased activation of PMN cells from RA patients. (A) Primary RA PMN were incubated with pre-fixed (1% paraformaldehyde) HTB-93 cells and collected at 0, 5, 15, 30 minutes and the lysate was run on a western blot and probed for the activity of ERK and AKT or total ERK/AKT as a loading control. (B) PMN cells isolated from the synovial fluid of RA patients and healthy blood donors were used in a cytotoxic assay with ^51^Cr-labeled HTB-93 cells. Percentage specific lysis of HTB-93 cells was compared using at effector to target (E:T) ratios of 50:1, 25:1,12.5:1, and 6.25:1. The mean and SD of triplicate determinations are shown.

Next we decided to further corroborate our hypothesis of increased PMN activity in RA through the analysis of cytotoxicity against a target cell line. For this purpose, we chose the human synovial cell line HTB-93 as our target cell after confirming them as useful targets by testing them against primary PMNs isolated from healthy donor cells and because we have had previous experience in killing assays with this particular cell line [22]. We cultured PMNs by themselves for 4 hours prior to admixing them with formalin fixed HTB-93 to measure the activation of ERK1/2 and AKT by western blot. Cell lysates were collected at 5, 15, and 30 minutes to visualize the kinetics of activation of normal PMNs after co-culture with inflamed synovia. There was a time-dependent increase in the activation of both ERK and AKT, although ERK itself decreased after its peak at 15 minutes (Fig. 3A). There was no increase in the overall levels of the total proteins in the lysates demonstrating an increase in activation after co-culture and the usefulness of HTB-93 as a target and inducer of inflammatory activation. When tested against the PMNs isolated from the inflamed synovia of RA patients, we found that these cells had an increased cytotoxic activity against these HTB-93 cells, compared to PMNs from normal specimens, corroborating that increased levels of activation through TREM1 correlate with the increased cytotoxic activity of PMNs from RA patients (Fig. 3B).

**Figure 4:**
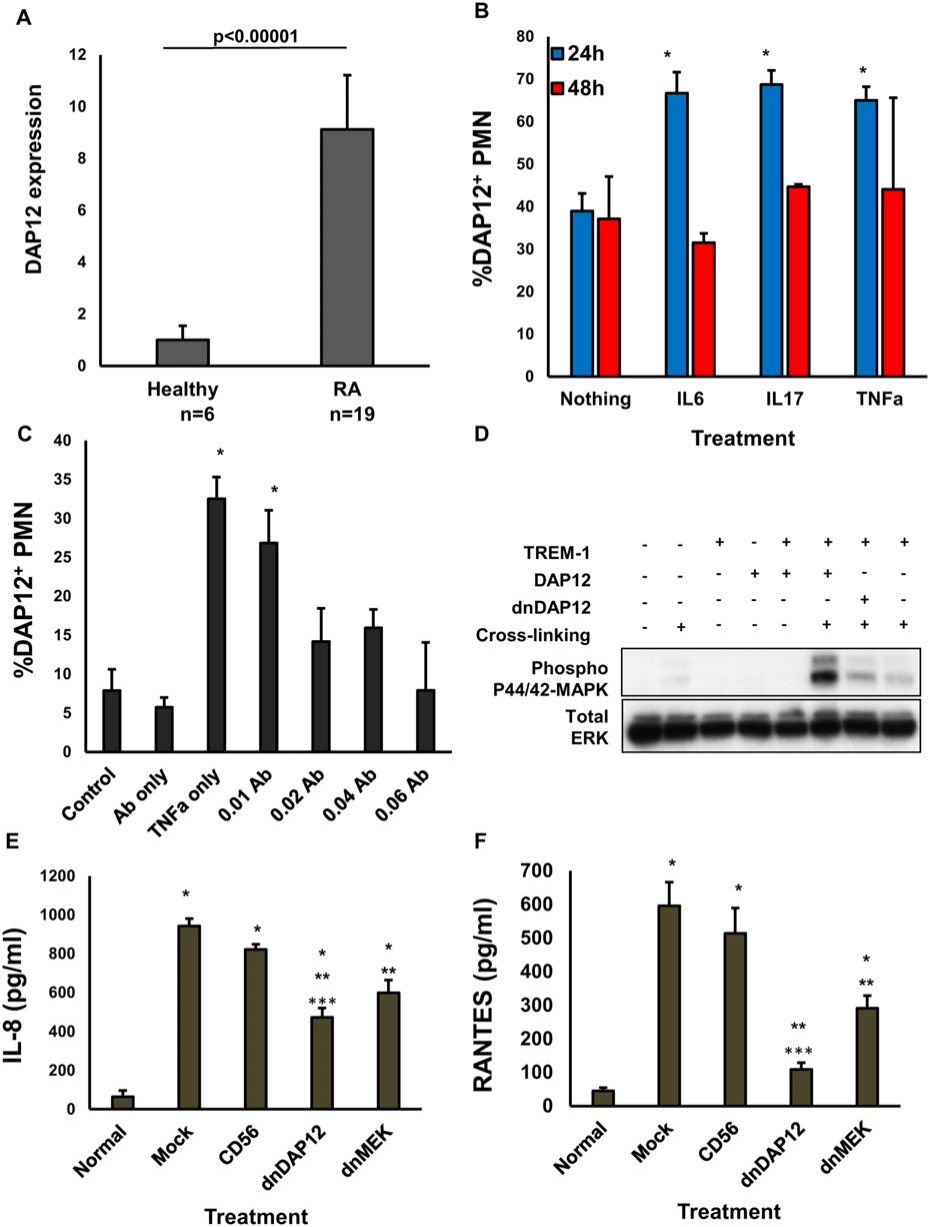
Blocking DAP12 reduces the increased signaling and cytokine production brought on by increased expression of DAP12 in PMN from RA patients. (A) Quantitative RT-PCR was used to determine the relative levels of DAP12 in isolated PMN from RA primary specimens (n = 19) compared with healthy controls (n = 6). Error bars denote the SEM of duplicate determinations per sample. P value shown. (B) Healthy PMN (n = 4) were isolated and cultured in the presence of either TNFα (1ng/ml), IL-6 (1ng/ml), or IL-17 (20ng/ml) for 24 and 48 hours before DAP12 analysis by flow cytometry. (C) Healthy PMN (n = 4) were isolated and cultured in the presence of TNFα after the addition of TNFα blocking antibody (0.01ug/ml to 0.1ug/ml) for 24 and 48 hours before DAP12 analysis by flow cytometry. For both B and C error bars denote the SEM of duplicate readings of 4 treated primary healthy specimens. Asterisk denote p<0.05 against control untreated cells. (D) HEK293 cells co-transfected with TREM-1 and either of the FLAG-tagged DAP12 constructs: wild type (WT, lanes 5, 6), or dnDAP12 (lane 7) were cross-linked with anti-TREM-1 antibody followed by western blot analysis for the activation of p42/p44 MAPK and compared against total ERK. Single transfections with TREM-1 and WTDAP12 are included as negative controls (lanes 3 and 4). Supernatants from this experiment were also used to analyze (E) IL-8 and (F) RANTES by ELISA were error bars denote the SEM of triplicate measurements of each sample and asterisk p<0.05 measured against * normal, ** Mock or ***CD56.

TREM1 only uses DAP12 as a signaling adaptor and it is also known to be increased in RA. Therefore, we studied if increases in TREM1 in RA were also met with an increase in the expression of this adaptor in the PMNs of RA patient primary specimens. Quantitative PCR of the isolated PMNs of primary RA samples demonstrates a significant increase in the gene expression levels of DAP12 (Fig. 4A, n = 6 control and n = 19 for RA specimens). Furthermore, similar to our observations with TREM1, DAP12 expression was also unregulated by culturing healthy PMNs with the inflammatory cytokines IL6, IL17 and TNFα (Fig. 4B) and blocking TNFα dose-dependently prevented the up-regulation of this adaptor (Fig. 4C) demonstrating a correlation between adaptor and receptor expression patterns in RA. We have recently reported the use of a dominant negative construct of DAP12 (dnDAP12) to study the role of this adaptor in the activation of myeloid-derived suppressor cells [23]. This mutant carries a tyrosine to alanine substitution at position 102, right within the ITAM domain rendering it inactive. To investigate if blocking TREM signaling through DAP12 would affect the activation of RA PMNs, we co-transfected 293T cells and cross-linked them with TREM1 antibodies and analyzed their activation by western blot. We observed that in the presence of wild type DAP12 there was an increase in MAP kinase activation which was avoided when transfecting with dnDAP12 or no activating molecule at all, demonstrating that DAP12 is necessary for TREM1’s signaling to occur (Fig. 4D). This observation was matched with a significant activation of IL8 (Fig. 4E) and RANTES (Fig. 4F) in RA specimens that was abrogated by the presence of the dnDAP12 adaptor.

**Figure 5:**
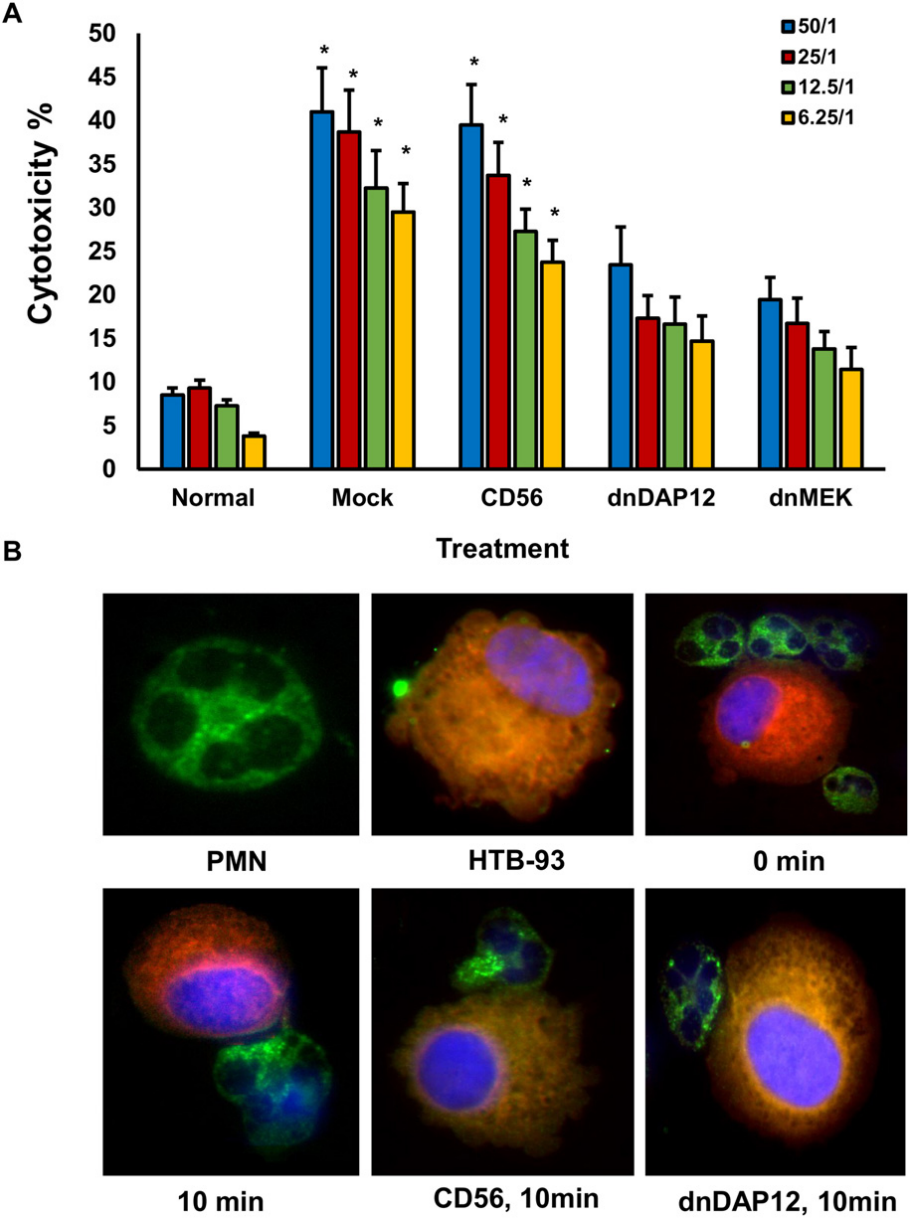
Expression of dnDAP12 in PMN cells from RA patients reduces adaptor signaling, cytotoxicity toward HTB-93 cells, and granule redistribution. (A) The cytotoxic properties of PMN cells from RA patients infected with recombinant vaccinia virus encoding CD56 (negative control), dnDAP12, or dnMEK (positive control) were admixed at an E/T ratio of 50:1, 25:1, 12.5:1 and 6.25:1 with HTB-93 target cells and evaluated in 5-hour ^51^Cr release assays. Mock-infected PMN cells from RA patients and healthy controls are included for comparison. The mean and SEM of triplicate wells per sample are shown (n = 5). Asterisk denote a p value of less than 0.05 against normal untreated cells. (B) PMN and HTB-93 cells were mixed at 1:1 E/T ratio and examined for granule redistribution. Green shows granzyme B, red shows pre-stained HTB target cells with Cell-Tracker Orange, blue reflects nuclear staining with DAPI. Results using untreated PMN RA cells are shown at zero and 10 minutes after mixing. Representative results using PMN RA patients cells expressing CD56 (negative control), dnDAP12 are shown. Representative experiment shown out of a total of 5 with similar results.

Functionally, transduction of primary PMNs isolated from RA patients with either empty vector virus (mock), CD56 vector (a negative control as it is not expressed by PMNs) increases its cytotoxic activity after co-culture with target cells (Fig. 5A). This effect was abrogated by transfection with either dominant negative MEK (a positive control) or our dnDAP12, demonstrating the important role of DAP12 in the activation of PMNs in RA inflammation (Fig. 5A). Importantly, dnDAP12 is also able to block granule mobilization to target contact zone as we observed a decrease in granzyme B granule concentration to the target contact zone after 10 minutes in dnDAP12 transfected cells by microscopic analysis (Fig. 5B).

## Discussion

PMNs, including neutrophils, which normally represent the first line of defense against bacterial infections, are also involved, through unknown circumstances, in the pathogenesis of RA [24]. In the present study we further support the important role that these activated cells play in this disease. Microscopically, rheumatoid joints are characterized by inflammatory infiltration of these cells in synovial fluid and the pannus-cartilage junctions where they are the first cells to infiltrate and contribute to cartilage erosion by releasing proteolytic enzymes and toxic oxidative products [25]. Overproduction of pro-inflammatory cytokines produced by macrophages, neutrophils and fibroblasts are also strongly associated with the pathology of RA where they are increased through either overabundance or insufficient inhibition at the joints [21, 26, 27]. TNFα, for instance, is a powerful mediator of the inflammatory response in RA through the activation of macrophages, the induction of other cytokines and even its capacity to make T-cells hypo-responsive with extended exposure [26]. The clinical implication is that, if the excessive activity of inflammatory mediators, such as TNFα, were blocked an overall reduction in inflammation would ensue both locally (in the synovial joints) as well as systemically, which is the target of many of the currently available agents for RA [25].

Despite the critical roles cytokines play in RA progression, treatments that block their function have proven to be only partially effective, indicating that other cytokines and pathways are involved in the disease [27]. As an example, a new class of agents, known as biologic response modifiers (BRMs), has been developed to block the effect of TNFα [28]. One of these agents is Etanercept (Enbrel), a genetically engineered protein containing two p75 soluble TNF receptors (sTNFRs) that are combined with the Fragment crystallizable (Fc) portion of the human IgG1 molecule [29, 30]. Etanercept has a higher affinity for the cell transmembrane receptors than the patient’s own endogenous sTNFRs and competitively inhibits TNFα from binding to the receptors thus reducing inflammation. However, even with drugs like this, or any of the available biological treatments like it, the majority of treatment responses comprise only 20% of patients, with most of those patients from early stage disease, leaving only 10–15% of those refractory to anti-TNF therapy responsive to any therapy [25, 31]. Because of the chronicity of the disease, even if a therapy is successful, long term adherence to therapeutic modalities remain in the 60% in the 1–2 year period dropping considerably after that mark, meaning that most patients will cycle through other therapies [25, 32]. Therefore, a deeper understanding of the biological mechanisms of disease pathogenesis in RA could provide a deeper understanding of the underlying causes of the disease as well as novel targets for the development of new, more effective, treatments and therapeutics.

Interestingly, many of those RA treatments, including glucocorticoids and TNF blockers, also inhibit neutrophil function which is probably one of the main contributors to the therapeutic efficacy of these agents [33]. Similarly, our studies show that an effect of TNF blocking is the down-regulation of TREM1 and DAP12 in PMNs, opening the opportunity to study other agents that can induce a similar function. Hence blocking of molecular signaling pathways that activate these cells could down-regulate the secretion of inhibitory cytokines as well as reduce the ensuing immune response. In particular, the cell surface activating receptor TREM1’s activation through DAP12 is not only highly up-regulated in RA PMNs but also play a key role in the development of the disease pathogenesis [16]. Its main function under normal circumstances is the activation of inflammatory conditions during infections caused by bacteria and fungi [10, 12]. Its expression is increased by activated toll-like receptors (TLR) during acute inflammation and, through crosslinking antibody on neutrophils and macrophages. It also expands the inflammatory signaling and increase the secretion of cytokines and chemokines, enhancing the immune response [34–37]. Importantly, it has recently been demonstrated that this receptor is highly expressed in CD14^+^ cells in chronic RA and in animal models, correlating with an increased production of the inflammatory cytokines TNFα, IL1β, GM-CSF and IL8 after stimulation of these cells in culture suggesting that blocking this pathway may be an important venue for the development of novel therapeutic strategies in RA [16]. Similarly, we here demonstrate that PMNs have a high expression of TREM1 which is not shared by other cells in the periphery, such as T cells and NK cells, and that these cells are highly functional as demonstrated by increased activation of both ERK and AKT as well as increased cytotoxic activity against the target synovial cell line HTB-93. Molad et al. demonstrated that TREM1 could be expressed in macrophages, even though we did not see this change in the more immature monocytes [8, 9]. These results confirm our observations as well as demonstrate that these cells are highly active in the synovium where they probably help in the induction of inflammatory damage to the joints.

TREM1 blockade has been tried already as a therapeutic option in sepsis animal models using either a chimeric molecule containing the extracellular domain of TREM1 and the Fc portion of a human IgG1 molecule or an antagonistic TREM1 peptide LP17, both of which reduced pro-inflammatory cytokines to sub-lethal levels [34, 38–40]. However, these are acute inflammatory models and a similar role has been described for TREM1 in other chronic inflammatory diseases, such as Crohn’s disease, ulcerative colitis and inflammatory bowel disease, through the use of agonist TREM1 [18, 41]. This allows it to serve as the basis to look at sTREM1 as an antagonistic therapeutic option for RA. An alternative which we suggest with the current experiments would be to target instead TREM1’ ITAM-containing adaptor DAP12, which we demonstrate has also an increased expression in RA. Using our recently reported dominant-negative DAP12 vector [23] on RA primary specimens, we observed a decrease in the activation of the pro-inflammatory signal ERK1/2 with a concomitant reduction in the production of pro-inflammatory cytokines. IL8 is an important chemokine that attracts neutrophils and basophils to the site of inflammation, and probably a key player in the amplification of the inflammatory response in RA supporting its crucial role in this disease, similar to RANTES [20, 26]. Both of these important cytokines were reduced by blocking DAP12 directly to healthy levels which means that in the least, blocking of DAP12 reduces the enhancement of the pro-inflammatory signaling produced by neutrophils. Importantly, this is also matched with a decrease in the functional activation of these cells through a reduction in the cytotoxic activity demonstrated earlier. Therefore, DAP12 could be considered a potential drugable target for RA to be studied either alone or in combination with currently available blocking antibodies or antagonistic peptides.

However, DAP12 is also an adaptor for other receptors that could be affected by such therapies starting with other TREM receptors such as TREM2 [42–47]. For instance, in addition to TREM1, another DAP12 associated receptor is the myeloid C-type lectin 5A (also known as MDL-1), which is abundantly expressed in neutrophils of RA patients and plays an important role in the pathogenesis of the disease [47, 48]. Moreover, activation of MDL-1 is able to cause bone erosion in RA in vivo, and inhibition of MDL-1 suppress collagen-induced RA [49]. However, these studies did not actively address the role of TREM1, which we show is up-regulated in RA patients, suggesting that other DAP12 receptors might also be involved in this disease’s pathogenesis. In a recent study, Crotti et al. demonstrated that TREM2 was highly expressed throughout tissues from active RA patients. Many types of immune cells including mononuclear cells in lymphoid aggregates appear to express TREM2 [8]. These receptors are highly expressed on immature dendritic cells (DC) and their engagement is known to help in their maturation as well as the transition from innate to adaptive immune responses. Recently, Bouchon et al uncovered the importance of the TREM family in the regulation of multiple facets of the immune response [34]. These studies defined TREM1 as an important mediator of septic shock and TREM2 as playing a unique role in dendritic cell maturation and, therefore, T-cell priming. Taken together, these data demonstrate the intriguing potential for receptors of the TREM family to be key regulators of both the innate and adaptive immune response. Importantly, other downstream effectors below DAP12-initiated signaling cascades such as JAK or SYK kinases have also been considered strong candidates for RA therapy using small molecules [25, 50, 51]. Importantly, these effectors are also part of other signaling pathways than TREMs but highly involved in the regulation of inflammatory immune responses whose end result again appears to be the overall down-regulation of cytokines and chemokines in RA. Unlike other treatments that focus on blocking specific cytokines, disrupting DAP12, and by extension, SYK which has also been shown to be up-regulated in RA [52, 53] and affects the action of receptors important for other immune cells such as B-cell receptors (BCR) and Fc receptors (FcR) on mast cells, macrophages, B cells and T cells [25].

In conclusion, RA is an important disease whose incidence is expected to escalate in the upcoming years, according to the US census, due to the increase in the life expectancy in the population. Importantly, current therapeutic strategies have been aimed at blocking the production of the main inflammatory cytokines involved in RA pathogenesis. However, current therapeutics are first not aimed at stopping the chronic inflammation and second not easy to maintain long term. Therefore, novel therapies that aim at down-modulating the main signaling pathways involved in the inflammatory response of RA could prove to be a novel venue with aims at more effective therapeutics.
